# Double-shot MeV electron diffraction and microscopy

**DOI:** 10.1063/1.4983390

**Published:** 2017-05-19

**Authors:** P. Musumeci, D. Cesar, J. Maxson

**Affiliations:** Department of Physics and Astronomy, UCLA, Los Angeles, California 90095, USA

## Abstract

In this paper, we study by numerical simulations a time-resolved MeV electron scattering mode where two consecutive electron pulses are used to capture the evolution of a material sample on 10 ps time scales. The two electron pulses are generated by illuminating a photocathode in a radiofrequency photogun by two short laser pulses with adjustable delay. A streak camera/deflecting cavity is used after the sample to project the two electron bunches on two well separated regions of the detector screen. By using sufficiently short pulses, the 2D spatial information from each snapshot can be preserved. This “double-shot” technique enables the efficient capture of irreversible dynamics in both diffraction and imaging modes. In this work, we demonstrate both modes in start-to-end simulations of the UCLA Pegasus MeV microscope column.

## INTRODUCTION

The time-resolved investigation of ultrafast processes typically relies on pump-probe schemes with an adjustable delay between the pump pulse and the probe. In typical ultrafast electron scattering experiments, the number of electrons per probe pulse is maintained very low in order to avoid the deleterious effects of space charge on the spatial and temporal resolution of the technique.^1–3^ The result is that in order to accumulate sufficient signal-to-noise to study a particular process, thousands to millions of shots have to be integrated, which constrains the realm of study to reversible phenomena.

For irreversible processes, the classical pump-probe approach where the delay is varied and the experiment is repeated many times to accumulate signal is not applicable. One costly and time-consuming solution is to maximize the number of electrons per pulse and utilize a fresh sample for each shot.^4^ However, there are cases when this may not be desirable. For example, in the study of defect dynamics, the specific size and type of defect may influence the evolution of the system. In this case, a pump and probe study where a new sample is used at each different delay will only lead to a measurement of a statistical ensemble which obscures the correlation between the local sample microstructure and its dynamics.

Continuously time-resolved ultrafast electron diffraction (UED), in which the electron beam is streaked after it interacts with the diffraction sample, is an interesting possibility to resolve this, as it adds temporal information to a single shot image. However, it is limited to processes which can be studied using one dimensional line-outs. For example, this has been demonstrated looking at the amplitude of the Bragg peaks of a gold sample upon laser excitation in a MeV UED setup^5^ and also in the ultrafast shadowgraphy study of electromagnetic field dynamics after laser-induced plasma formation.^6^

A different approach is required when 1D information is insufficient, such as in direct imaging or when diffracting from a polycrystalline sample. At the cost of losing most of the signals, in the latter case, in principle, it is possible to use a slit to streak a line-out of the diffraction pattern. Here, we proposed a solution inspired by the recent advent of two pulse or two color schemes at X-ray free electron laser facilities. There are different ways to obtain two separate x-ray pulses after the undulator. For example, beam manipulation at high energy can lead to the formation of two electron pulses at slightly different energies.^8^ One of the simplest arrangements is to temporally split the laser pulse illuminating the cathode so that two fully independent electron beams can be generated and transported to the undulators.^7,9^

A similar scheme has been introduced in electron scattering machines in the movie mode dynamic transmission electron microscope (DTEM)^10^ where an arbitrary laser waveform generator and two deflecting plates were used to project up to 16 different snapshots of a process in different regions of the detector screen. Here, we explore the possibility of applying this technique to MeV UED and ultrafast electron microscopy (UEM) with radiofrequency photoinjectors, which offer significant advantages in the suppression of space charge forces and penetration depth, among others. A particular application where this will be useful is the study of defect motion.^11^ Dislocations can be imaged in an electron microscope with relatively low spatial resolution using diffraction contrast. Their motion determines all the mechanical properties of the materials and is therefore highly important. A double-shot illumination will be able to provide to consecutive snapshot of one of these defects allowing to greatly enhance our understanding of the underlying dynamics.

**FIG. 1.**
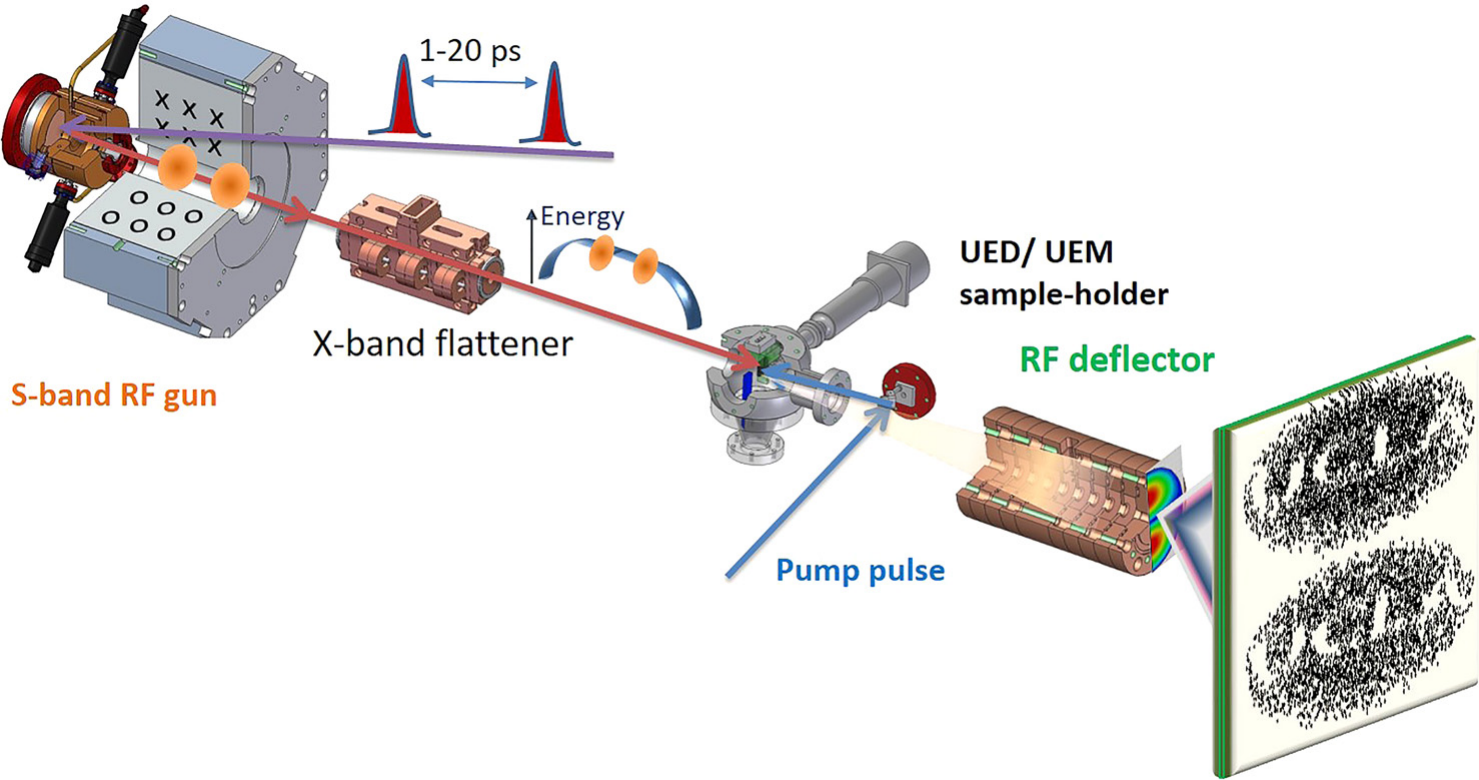
Experimental layout for the simulated double shot electron scattering mode. Two adjustable delay ultrafast photoemission pulses generate two separate electron bunches in a 1.6 cell S-band RF photoinjector, which is followed by a round solenoid electron lens, an RF linearizing cavity, the sample interaction point, and an RF deflecting cavity with the final detection screen.

The scheme is shown in Fig. 1. Two electron beams are generated by illuminating the cathode with two laser pulses with adjustable delay. An RF streak camera is used to separate the electron beams at the detector after interaction with the sample. The primary technical challenge of this approach is due to the rapidly varying nature of the RF fields, which places an upper bound of the temporal separation of two pulses. For instance, one degree of RF phase at S-band frequencies corresponds roughly to one picosecond. A separation of the two pulses on the order of 10 ps thereby introduces a significant energy and chirp mismatch between the two beams.

This mismatch can be mitigated by using a linearizing RF cavity with higher frequency than the gun, which can flatten the output energy profile as a function of bunch arrival time. This is the solution explored in this work. In particular, this case is modeled on the UCLA Pegasus beamline^12^ where the addition of a linearizing cavity in the X-band (9.6 GHz) will provide a flat energy region of up to 20 ps. Nevertheless, the concept presented here can be easily generalizable to different setups. In particular, longer wavelength radiofrequency guns (such as the VHF Apex gun^13^ or a 200 MHz quarter-wave resonator superconducting RF gun) will have the advantage of allowing a longer allowable delay between the two pulses.

The paper is organized as follows: we describe the generation of electron beams with tunable delay in an RF gun and linearizer geometry. We first apply this method to double-shot ultrafast electron diffraction (UED). We then discuss detailed simulations of double-shot ultrafast electron microscopy (UEM) using two magnification stages composed by permanent magnet quadrupole based lenses.

## TWO BEAM GENERATION

It is straightforward to generate two ultrashort laser pulses with a variable delay, for example, using beamsplitters and a Mach-Zender type interferometer configuration.^14^ It is important that the separation between the two pulses be much larger than their pulse length, so that during subsequent deflection, the smearing in the streaking direction of the individual pulses is minimized. The bunch length of each sub-pulse (*δt*_0_) and their separation at the deflector (Δ*t_sep_*) must then obey

$$\frac{\delta t_{0}}{\Delta t_{sep}}\leq \frac{R}{S},$$ (1)

where *R* and *S* are the desired spatial resolution and the separation of the two bunches on the final screen respectively, both evaluated along the streak direction.

The RF linearizer used in our simulation is a side-coupled X-band structure and is illustrated in Fig. 1. Currently under construction, this cavity will be installed 60 cm downstream the gun in the UCLA Pegasus Laboratory. Beyond enabling this double shot mode, the cavity will also allow full longitudinal phase space linearization and bunch compression.

The linearizer consists of a 7-cell accelerating section, with a relative phase velocity matching the gun exit velocity, *β* = 0.9922. The beam pipe radius is 4 mm, and accelerating cell noses are added to increase the accelerating voltage. Coupling cells are required to make the structure operate in the *π*/2 mode, which has larger frequency separation with neighboring modes than the *π* mode and thus increases the fabrication tolerances of the structure. The accelerating structure is designed to deliver an accelerating voltage of 300 kV with the input power less than 10 kW.

**FIG. 2.**
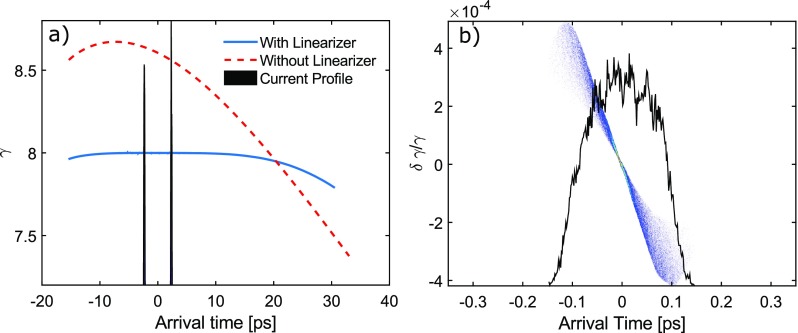
(a) Energy vs. launch time. The flattened profile is the result of the X-band linearizer. The current profile of the two beam pulses is also shown. (b) Longitudinal phase space of the first bunch, with the current profile overlaid in black in arbitrary units.

By requiring a null second derivative for the total energy, it is easy to show that the extent of the flat region is maximized when the energy imparted by the x-band cavity is equal to (*f*_1_/*f*_2_)^2^ times the output energy from the RF gun and the relative phase shift is equal to *π*. In this expression, *f*_1_ = 2.856 GHz is the frequency of the gun and *f*_2_ = 9.6 GHz is the frequency of the linearizer. The energy output of the linearizer as a function of arrival time is shown in Fig. 2, which illustrates a 20 ps long flat energy region, defined as the maximum temporal width where the energy is constant up to 0.1%. While the synchronization jitter between the gun and linearizer RF signal is an important parameter, a relative jitter of 500 fs (which should be easy to obtain using standard low level RF techniques), only reduces the flat energy region to 18 ps.

While the centroid energies of the two bunches are nearly identical, the relative energy spread *δγ*/*γ* of each of the two beams can still be ∼10^–4^. Such a low energy spread is not a critical parameter for diffraction experiments nor for direct imaging experiments, as we find that in single-shot time-resolved electron microscopy, the effects of chromatic aberrations are smaller than the resolution limit set by space charge effects as discussed in Ref. 18.

Start-to-end simulations of the beam dynamics for both diffraction and imaging in the Pegasus beamline are carried out using the General Particle Tracer code.^15^ The beamline parameters are listed in Table I.

**TABLE I.** Parameters for double-shot electron scattering beamline.

Charge per pulse	10–20 fC
Distance	1–20 ps
Laser spot size at cathode	30 *μ*m
Beam energy	4.1 MeV
Laser pulse length	40 fs
Initial emittance	12 nm

## DIFFRACTION

The first case we analyze is the diffraction configuration. In this case, after the sample there is no beam element other than the X-band deflector, which is used to spatially separate the beams. In the simulation of the diffraction pattern, we assume diffraction from a 100 nm thick aluminum polycrystalline sample. In this case, the diffraction pattern will have the characteristic ring-like structure so that it might be desirable to preserve the 2D spatial information—i.e., it cannot be characterized by 1D projected image.

When tracking particles in the deflector, a full model for the deflecting cavity fields from a 3D electromagnetic simulation code is used. Though the deflection cavity is capable of operating at a deflecting voltage of *V_d_* = 500 keV and can have resolution in the sub-10 fs range,^12^ considering the relatively long time separation of the two bunches, this case offers greater potential for image preservation in the streaking process. The angle between the two bunches after they pass through the deflector is given by

$$\Delta y\prime =S/L=\frac{eV_{d}}{mc^{2}\gamma }\omega \Delta t_{sep},$$ (2)

where *L* is the distance from the deflector to the final screen, *ω* is the deflecting cavity angular frequency, and *e*/*m* is the charge to mass ratio of the electron. The Bragg angle is *θ_B_* = *λ*/*d*, where *λ* = *h*/*p* is the wavelength of the electrons and *d* is the atomic plane separation, and this angle determines the deflecting voltage strength. For example, for Al(200), *d* ≈ 0.2 nm, corresponding for 5 MeV electrons to a Bragg angle *θ_B_* = 1.5 mrad. To ensure that the diffraction rings are separated, we require $\Delta y\prime >\theta_{B}$, which requires only *V_d_* ranging from 10 to 200 keV for Δ*t_sep_* from 1 to 20 ps. At these small deflecting fields, image distorting effects such as deflector defocusing (which scales as $V_{d}^{2}$) are greatly reduced.^17^

We can use the spacing of the Bragg peaks to set the scale for the desired spatial resolution at the detector plane *R*. While the beam coherence, as given by the initial emittance, is sufficient to resolve closely spaced Bragg peaks, we choose the bunch length *δt*_0_ to be short enough that they do not overlap when streaked. Assuming that the scale of the peak separations is *R* ∼ *Lθ_B_*/10 (which is approximately the separation of the [200] and [111] peaks), we arrive at the condition that *δt*_0_ < Δ*t_sep_*/10. This condition is easily satisfied by electron bunches generated by ultrafast Ti:Sapphire pulses, typically on the order of 50 fs rms bunch length, which suggests a degree of preservation of spatial information in the streaked final image.

The high frequency deflecting cavity naturally has small transverse dimensions, with a clear aperture of only 1 cm diameter. Therefore, it must be placed close to the sample to minimize particle loss as the beam expands after diffraction. In our case, the distance between the sample and the deflector is <20 cm. This also has the added benefit of minimizing deleterious effects from the deflector such as induced energy spread (a consequence of the Panofsky-Wenzel theorem) and field nonlinearities, as these both grow with increasing vertical beam size in the cavity. Note that in the above estimates, it was not necessary to specify the distance from the sample to the detector. This distance can be chosen to provide sufficient k-space resolution from the detector. It may be beneficial to use large area detectors to capture the double image, which is easily accomplished with large phosphor sheets.

**FIG. 3.**
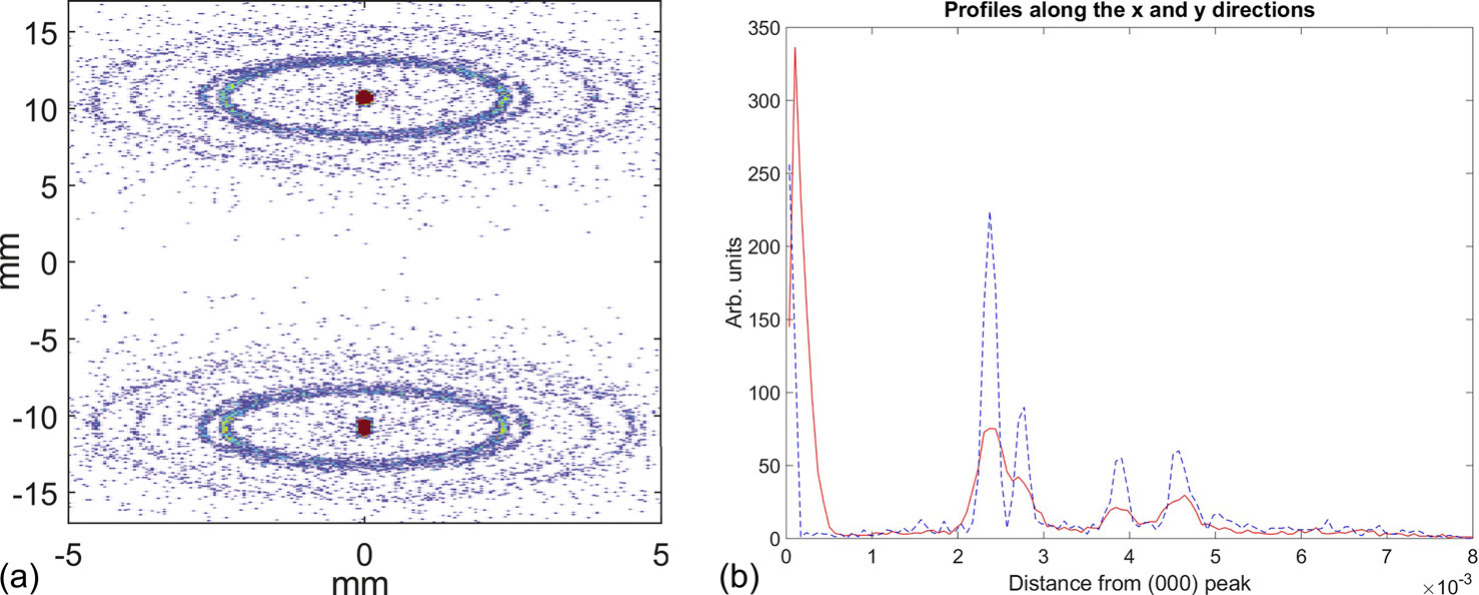
(a) Simulated diffraction pattern from polycrystalline Al on the final detector, in the double shot mode. Note the axis break. The diffraction patterns result from two nearly identical bunches displaced in time by Δ*t_sep_* = 12 ps. These two bunches are then displaced vertically with a transverse deflecting cavity. As the individual bunch lengths are much shorter than their separation, there is limited smearing of each pattern vertically. (b) Comparison of line-outs in the streaking (red) and non streaking (blue, dashed) directions.

The start to end simulation is performed assuming smooth space charge. At the diffraction sample, the distribution of momenta is reorganized according to the likelihood of scattering into the first five Bragg peaks of polycrystalline Al. After passing through the deflector, the particle transverse positions are read out at a screen corresponding to the final detector. The resultant double pattern is shown in Fig. 3.

## IMAGING WITH HIGH MAGNIFICATION

We now present simulations of a case in which a real image is formed on the final detector screen using two magnification stages. Imaging at relativistic energies requires extremely strong magnetic lenses, and given the unfavorable scaling of the focal strength of solenoids with energy (*f* ∼ *γ*^2^), quadrupole triplet magnification stages (which have *f* ∼ *γ*) have been proposed and recently demonstrated at UCLA.^16^ However, in that first experiments only one triplet was used, limiting the magnification to 35×.

Here, we present the results of simulations for two stages, which will provide a magnification of *M* = 1000×. Given the ability to image the final detector fluorescent screen with near micron resolution, this magnification holds the promise of ultrafast electron microscopy with 10 s of nanometer spatial resolution in conjunction with ps temporal resolution.

**FIG. 4.**
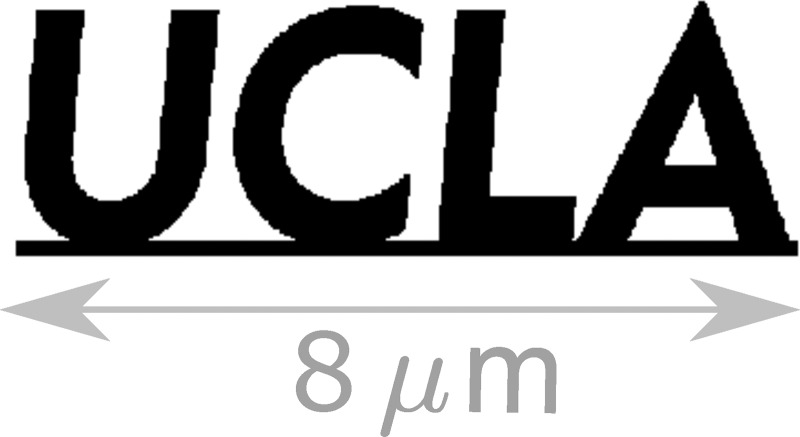
The geometry of the target used for mass contrast imaging is shown in black, and a scale bar is shown in gray. In order to calculate the effect on the electron beam (angle scattering and energy loss), the sample is assumed to be a 20 *μ*m thick Cu.

For the first time, this two stage simulation is performed start-to-end, where dynamics are tracked from the photoemission process to the final detector, and including the relevant binary electron interactions. Each of the two stages used here is a copy of that used in Cesar *et al*.,^16^ separated by 30 cm. The target used in the simulations is shown in Fig. 4, where in simulation we suppose it to be made of 20 *μ*m thick copper. The imaging is provided by mass contrast and in simulation the portions of the beam which pass through the sample are given a normally distributed random angle consistent with 5 MeV electrons scattering off a thick piece of copper. Collimation of target-scattered particles is performed with an aperture of radius 100 *μ*m at the position of the first triplet stage. A second collimation occurs due to the finite deflector iris, where the deflector is located 10 cm downstream of the second triplet. The final image plane is located 1 m downstream of the sample.

We can again estimate the bunch separation, deflecting voltage, and vertical smearing of the final image. The length scale of the target is *H* = 8 *μ*m, and hence, *S* = *MH* = 8 mm. A 5 ps bunch separation is used in the imaging simulations, and thus, the required deflecting voltage is *V_d_* = 200 kV. For a rms bunch length of *σ_t_* = 40 fs, this implies a deflector-induced blurring on the final screen of 80 *μ*m or 80 nm in imaging resolution. This has been confirmed by comparing simulations with and without the deflecting cavity.

It has been previously shown that fundamental resolution limitations arise from stochastic electron interactions (as opposed to smooth space charge), which become important when considering the very strong cross-over in the imaging process. To model this effect, we track individual electrons (20 fC total) from the photocathode to the sample using a mesh-based Poisson solver (smooth space charge). It is possible to neglect stochastic interactions up to the sample, as the lack of strong foci makes this portion of the transport well described by a mean field model. Furthermore, it is only the Coulomb induced scattering after the sample which determines the image quality. Therefore, only at the sample and beyond do we turn on stochastic interactions using the GPT integrated Barnes Hut method.

**FIG. 5.**
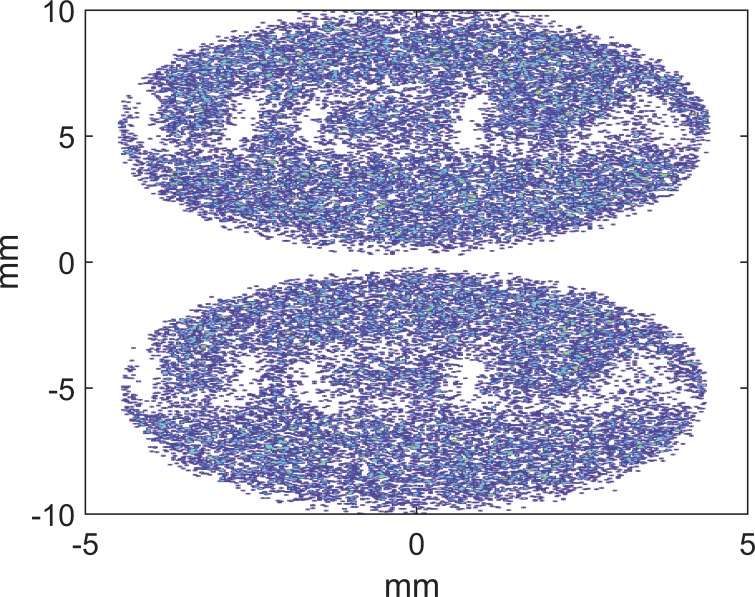
Double shot image of the scattering target on the final detector screen including stochastic electron interactions. The pixel size is 25 *μ*m, and an additional 25 *μ*m rms point spread function for the detector is assumed. The units on the axes are millimeters as measured on the final screen or microns at the sample, as the design magnification is 1000×.

The final image on the detector screen after passing through the deflector is shown in Fig. 5. A best fit for the magnification factor can be obtained comparing the particles' coordinates with their position at the sample plane and is found to be ∼1080 in good agreement with the space-charge off design. The blurring of the final image induced by the Coulomb interaction is limited and allows to well resolve the 100 nm scale features in the original target for both electron pulses.

## CONCLUSIONS

In conclusion, in this paper we have analyzed the possibility of using two consecutive electron pulses to obtain consecutive snapshots of the sample at an adjustable time-delay between 1 and 20 ps. In principle, more than two pulses can be used even though for multiple illumination the challenge would be to optimize pulse durations and relative spacing to minimize the smearing in the vertical resolution of the final image. For example, using a larger detector screen, it will be easily possible to use three bunches spaced by 5 ps instead of the two used in the simulations presented here to obtain a three frame illumination.

Another possibility if a longer time-window is being studied would be to use multiple pulses spaced at the periodicity of the accelerator. For a purely S-band RF system, this temporal distance would be 350 ps. In our setup, the x-band linearizing cavity is driven at ∼9.6 GHz which is not a simple multiple of the S-band frequency so that the minimum spacing of the pulses should be 1.05 ns for the electrons to have the same dynamics. It would be interesting to apply this technique to other MeV electron sources which have a flatter output energy profile than the S-band RF photoinjector and could potentially accommodate a larger number of well separated pulses.

This paper also shows for the first time detailed start-to-end simulations of a two stage imaging system for relativistic electrons. The design will be tested at the UCLA Pegasus Laboratory to demonstrate single-shot 10–100 nm spatial resolution with ps-long bunches.

Finally, it should be mentioned that the current work can be seen as an extension of the continuously time-resolved streaked UED technique to solve the problem of loss of two dimensional information (for example, due to overlapping Bragg peaks). A number of algorithms, borrowed from the fastly evolving compressed sensing field,^19^ could also be applied to invert the problem of streaking and reconstruct the original 2D image. On the other hand, the idea discussed here of using well separate electron beams is a more direct approach to preserve the 2D information in streaked electron scattering experiments.
